# Identification and Validation of Urea Transporter B Inhibitor from *Apium graveolens* L. Seeds In Vitro and In Silico

**DOI:** 10.3390/molecules30071540

**Published:** 2025-03-30

**Authors:** Guanzhong Chen, Xin Li, Xinhui Pan, Li Guo, Wei Wei, Xiaoying Sun, Hongtao Wei, Xue Qin, Ke Zhang, Wei Zhang, Lili Wei, Pinghua Sun, Xiaoda Yang

**Affiliations:** 1 School of Pharmacy, Key Laboratory of Xinjiang Phytomedicine Resource and Utilization Ministry of Education/Institute for Safflower Industry Research, Shihezi University, Shihezi 8320002, China; cgz970718@163.com (G.C.); gl20180108@163.com (L.G.); junoww@163.com (W.W.); s17731511105@163.com (X.S.); 18265526516@163.com (H.W.) xjzk1984@163.com (K.Z.); zhangwei_1994@shzu.edu.cn (W.Z.); pinghuasunny@163.com (P.S.); 2 Stake Key Laboratory of Natural and Biomimetic Drugs, Department of Chemical Biology at School of Pharmaceutical Sciences, Peking University, Beijing 100191, China; lx15264024579@163.com; 3 The Key Laboratory of Xinjiang Endemic and Ethnic Diseases, Ministry of Education, Shihezi University Medical College, Shihezi 832000, China; 17852425027@163.com (X.Q.); wll1126@shzu.edu.cn (L.W.); 4 International Cooperative Laboratory of Traditional Chinese Medicine Modernization and Innovative Drug Development of Chinese Ministry of Education (MOE), College of Pharmacy, Jinan University, Guangzhou 510632, China

**Keywords:** urea transporter inhibitors, diuretic, celery seeds

## Abstract

Celery (*Apium graveolens* L.) seeds are rich in carbohydrates and protein, and they are widely used in diuretic drugs among Uyghur doctors. However, the diuretic mechanism is still unclear. To explore the possible diuretic mechanism of celery seeds, urea transporters, a potential diuresis-related target, are used in this study. Urea transporters (UTs) play a key role of urine concentration. Selective knockout of UTs can concentrate urea without affecting water and electrolytes, resulting in selective diuresis, which is a promising new diuretic target. In the present study, we obtained different polar fractions by extracting and separating celery seed extract, characterized its polar fractions using UPLC-TOF-MS, and verified its action using an erythrocyte lysis model in vitro. Then, it was found that the isovaleric acid *p*-tolylester exhibited moderate activity (IC_50_ = 80.34 μM). Finally, its inhibitory effect on UT-B was investigated by using molecular docking, a pharmacophore model, and molecular dynamics simulations. This study provides a new approach to developing novel diuretics.

## 1. Introduction

Diuretics are mainly used to treat edema, heart failure, liver cirrhosis, hypertension, and nephrotic syndrome [1]. Conventional diuretics, such as loop diuretics, thiazide diuretics, potassium-conserving diuretics, and carbonic anhydrase inhibitors, produce diuretic effects by directly or indirectly inhibiting Na^+^ reabsorption, which can cause electrolyte disturbance with long-term use. Subsequently, it will affect the healthy conditions of the body, such as through hypokalemia, arrhythmia, and even life-threatening complications [2]. Therefore, there is an urgent need for novel diuretics that can be taken long-term and have fewer side effects.

Urea transporters (UTs), which are a kind of membrane channel protein, facilitate urea transmembrane permeability [3]. UTs include two sub-families: UT-A and UT-B. UT-A and UT-B are encoded by *Slc14a2* and *Slc14a1* [4,5]. In the kidney, UT-A1 and UT-A3 are expressed in the collecting ducts [6]. UT-B is expressed in the descending vasa recta and other tissues, such as erythrocyte, brain, heart, colon, testis, bladder, etc. [7]. UTs play a key role in the process of urea concentration [8]. UT-B knockout mouse models [6] demonstrate that the lack of UT-B leads to a diuretic effect without causing electrolyte imbalances, and the urine output of UT-A1 knockout mice is approximately three times that of wild-type mice. The urea concentration in the inner medullary tissue fluid of these knockout mice is significantly lower than that of wild-type mice, while the concentrations of Na^+^, K^+^, and Cl^−^ show no significant differences compared to wild-type mice. In terms of growth and development, renal function indicators, blood electrolyte levels, and renal tissue structure, UT-A1 knockout mice exhibit no significant abnormalities [5], which indicates that UTs hold promise as novel diuretic targets [9]. However, UT-B inhibitors have been found to cause an increase in blood urea concentration in mice [7], and the accumulation of urea in the hippocampus can lead to depression-like behaviors [10,11]. Additionally, the accumulation of urea in the testes is associated with premature maturation of the male reproductive system. Furthermore, UT-B knockout can induce DNA damage and apoptosis in bladder urothelial cells [12,13]. Due to the high structural similarity between UT-A and UT-B [14] and the fact that UT-B is widely distributed while UT-A is expressed only in the kidneys, the screening of UT inhibitors is typically conducted through in vitro assays targeting the more ubiquitously present UT-B. Therefore, UT-B is more suitable for high-throughput in vitro screening, while UT-A inhibitors are more suitable for development as novel diuretics.

**Figure 1:**
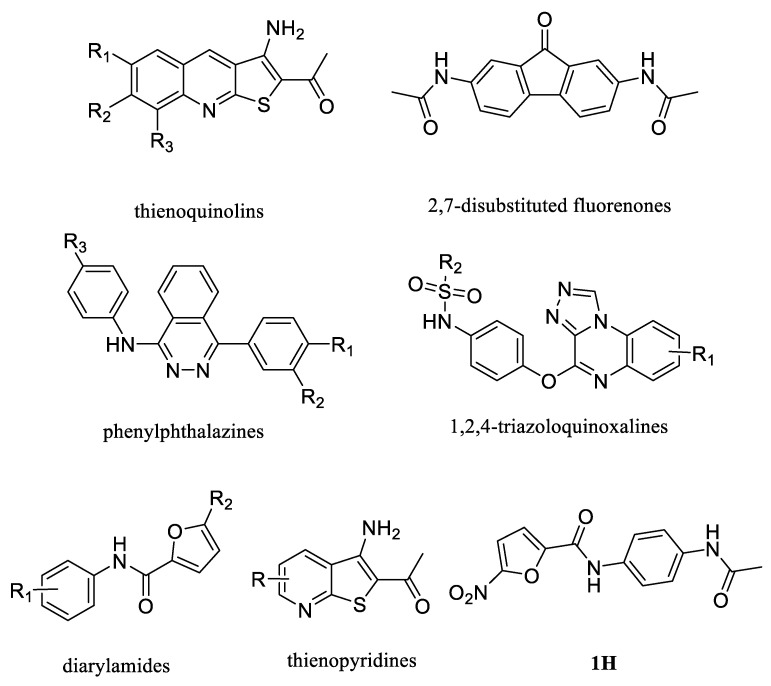
Molecular structures of reported UT-B inhibitors.

In recent years, several UT inhibitors have been reported. The molecular scaffold is also diverse, including thienoquinolin [15], 2,7-disubstituted [16], phenylphthalazines [17], 1,2,4-triazoloquinoxalines [18], and diarylamindes [19,20] (Figure 1). In particular, the diarylamides of Yang’s group, **1H** [19], showed excellent UT inhibitory activity at the sub-micromolar level in vitro and good oral bioavailability in vivo. And, according to Zhang’s experiments [19], the administration of UT inhibitors over a continuous period of 7 days significantly increased urine output in rats without causing side effects, such as electrolyte imbalance. Additionally, Ying’s experiments [21] demonstrated that the UT inhibitor can slow down the development of ascites induced by dimethylnitrosamine (DMN) in liver cirrhosis.

Most of the currently reported UT inhibitors are obtained by screening small molecular libraries. However, there are few reports on the search for UT-B inhibitors from natural products as novel diuretics. In this research, we aim to search for some small molecules with good UT-B inhibitory activity and high bioavailability from natural products and use computational methods for validation.

Celery seeds are the seeds of the celery plant (*Apium graveolens* L.) from the Umbelliferae [22]. Celery seeds are mainly used in the treatment of hypertension, arthritis, rheumatoid arthritis, pneumatosis, ascites, and kidney diseases. Fresh and dried celery have nervine, stomachic, appetite stimulating, wind expelling, detoxifying, and emmenagogue properties [23]. According to a literature research, celery seed contains three phenolic compounds, one glycoside, five flavonoids, four volatile oils, 12 terpenes, 21 fatty acids (or esters), and 30 compounds belonging to other categories [24,25,26,27,28,29,30,31,32,33] (Table S1). Celery seed is consumed as a diuretic; it is also used to treat symptoms of kidney, rheumatoid, and arthritis diseases [34]. The study by Maryam Hassanpour Moghadam demonstrated that celery seed extract reduces blood pressure and heart rate in hypertensive rats induced by deoxycorticosterone acetate [35]. Furthermore, Jun Zhu’s research indicates that 3-n-Butylphthalide (NBP), extracted from celery, may slow the advancement of hypertensive nephropathy by mitigating tubular damage, significantly reducing oxidative stress, and decreasing the expression of proinflammatory cytokines and TGF-*β*1 in kidney tissues [36]. Additionally, Maryam Shayani Rad’s study presents a randomized, triple-blind, placebo-controlled, crossover clinical trial involving hypertensive patients who consumed celery seed extract capsules. The findings suggested that celery seed extract can be regarded as a safe supplement for hypertensive patients without affecting renal biochemistry function or serum electrolytes [37]. However, these studies have primarily focused on the NBP extracted from celery seeds or the crude extract of celery seeds. Our aim is to explore other substances within celery seed extract and their novel modes of action, offering new possibilities for research on celery seeds. Because all currently known diuretics exert their diuretic effect by affecting sodium ion concentration, while UT diuretics do not affect electrolyte concentration, and celery seeds also do not affect electrolyte concentration when exerting their diuretic effect, we hypothesize that celery seeds may exert their diuretic effect by acting on UT-B. Therefore, this study aims to screen and validate novel UT-B inhibitors in celery seeds using an erythrocyte lysis model, molecular docking, pharmacophores, and molecular dynamics simulations in natural products.

As a membrane channel protein, UT poses challenges for experimentally validating its interactions with small molecule inhibitors. However, the rapid advancement of computer technology enables us to study these interactions in silico. Computational methods have been widely used to understand and predict the potential for ligand and protein target interactions [38], including structure–activity relationship (SAR) analysis, pharmacophore, molecular docking, molecular dynamics, and homology modeling studies [39], and, to some extent, these approaches can help us better interpret experimental results, reduce experimental time, and predict pharmacokinetic parameters and drug toxicity. Pharmacophores are the collections of spatial and electronic features that are required to ensure optimal supramolecular interactions with specific biological target structures and to activate (or inhibit) their bioactivity [40,41,42]. This provides significant assistance for the development of novel UT-B inhibitors. For example, by utilizing molecular docking technology and pharmacophore models, we can rapidly screen for the characteristic elements and modes of action through which UT-B inhibitors exert their biological activity. Additionally, ADME predictions can help assess the ADME properties of compounds, enabling the early exclusion of small molecules with poor ADME characteristics.

## 2. Results and Discussion

### 2.1. The Activity of Different Polarity Fractions In Vitro

**Figure 2:**
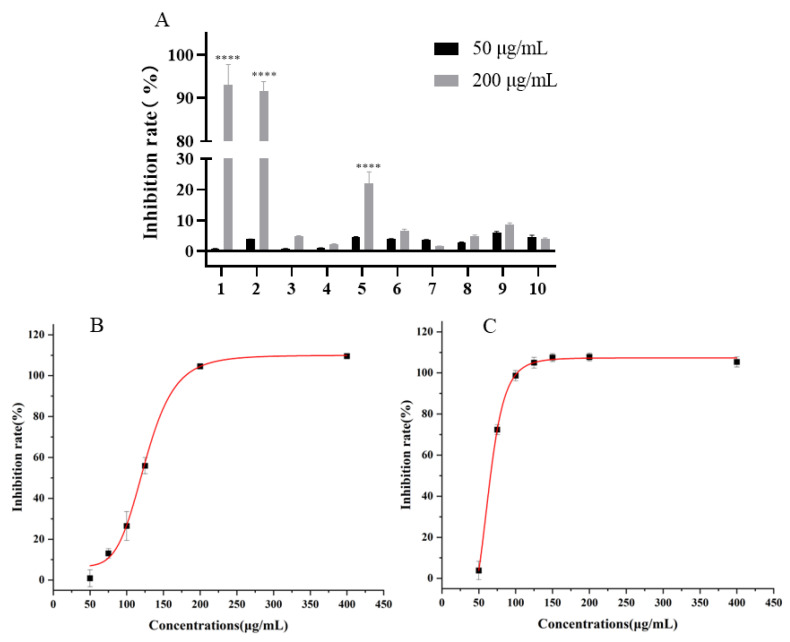
The active fractions of celery seed. (**A**) Inhibitory activity of celery seed column chromatography fractions against UT-B. (**B**) The erythrocyte lysis model measuring fraction 1 inhibition. R^2^ = 0.99269; IC_50_ = 119.59 ± 3.12 μg/mL. (**C**) The erythrocyte lysis model measuring fraction 2 inhibition. R^2^ = 0.99667; IC_50_ = 65.93 ± 0.74 μg/mL. Data are means ± SEM; *n* = 3. **** *p* < 0.0001, compared with 50 μg/mL.

By changing the ratio of dichloromethane to methanol and utilizing gradient elution, we obtained a total of 10 different fractions. These fractions were screened for activity by using the erythrocyte lysis model in vitro, and the results are shown in Figure 2.

As shown in Figure 2, it can be seen that the active fractions of celery seed were fraction 1 (IC_50_ = 119.59 ± 3.12 μg/mL) and fraction 2 (IC_50_ = 65.93 ± 0.74 μg/mL). However, the dose–response relationship for fraction 2 was not robust. Thus, fraction 1 and fraction 2 were used for subsequent studies. Moreover, this is the first time that fractions with UT-B inhibitory activity have been identified in celery seeds in vitro.

### 2.2. Characterization of the Constitutions of Apium graveolens L. Through HPLC-TOF-MS

**Figure 3:**
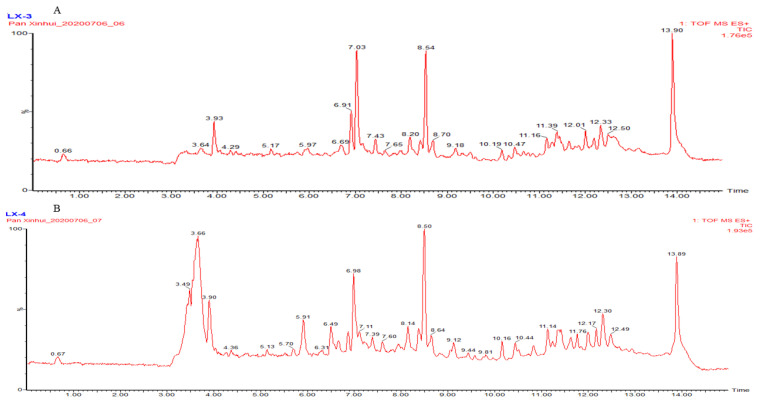
Base peak chromatogram from *Apium graveolens* L. extract obtained in positive ion mode. (**A**) *Apium graveolens* L. extract fraction 1; (**B**) *Apium graveolens* L. extract fraction 2.

The two fractions, fraction 1 and fraction 2, which exhibited good inhibitory activity against UT-B in vitro, were selected for UPLC-TOF-MS analysis. Through comparison with the compound library, the following 10 major compounds were identified (Figure 3, Table 1 and Table 2)

Using UPLC-TOF-MS technology, we identified a total of eight major chemical components from the two active fractions, which are Isovaleric acid *p*-tolylester, carvacrol, Cinene, Valeric acid, Allyl phenyl ether, *p*-Hydroxybenzaldehyde, linolenic acid, and *β*-Myrcene. Subsequently, we conducted in vitro activity screening for these eight compounds.

### 2.3. Inhibition Activity of Compounds Against UT-B

**Figure 4:**
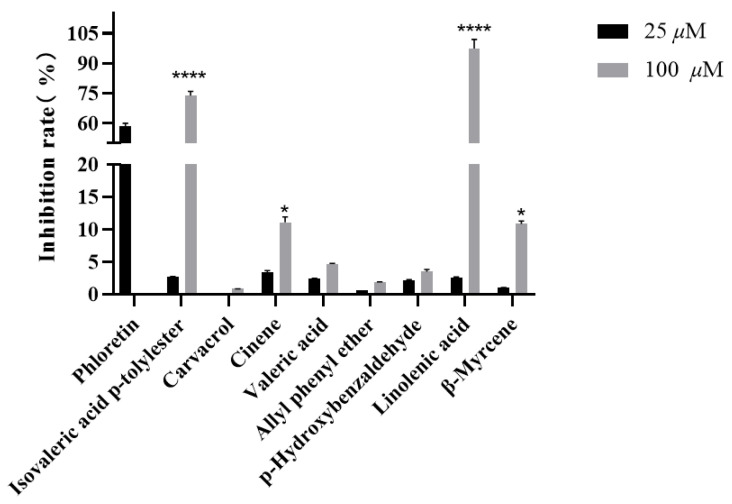
UT-B inhibitory activity of compounds in celery seed fractions 1 and 2 detected through UPLC-TOF-MS. Data are means ± SEM; *n* = 3. * *p* < 0.05, **** *p* < 0.0001, compared with 25 μM.

After purchasing the eight main compounds for in vitro activity screening, these eight compounds were subsequently screened for in vitro activity using the erythrocyte lysis model. The result is shown in Figure 4.

**Figure 5:**
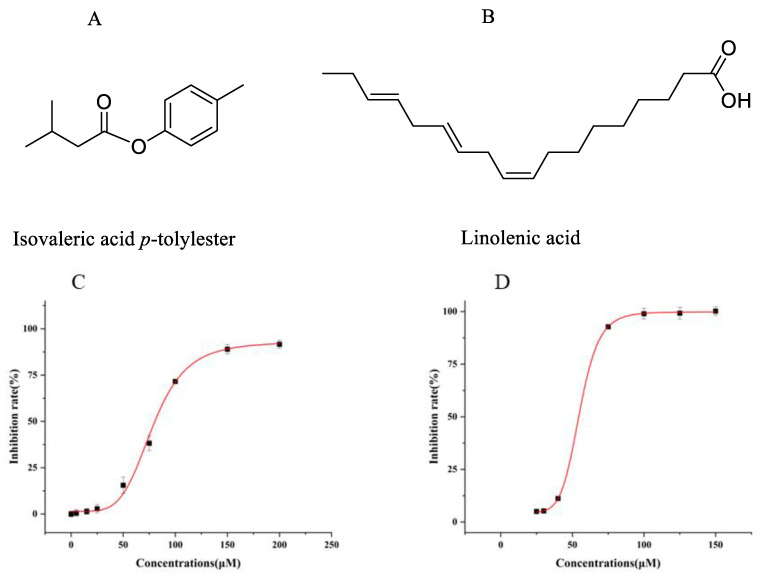
The erythrocyte lysis model measuring isovaleric acid *p*-tolylester and linolenic acid inhibition. (**A**) The structure of isovaleric acid *p*-tolylester. (**B**) The structure of linolenic acid. (**C**) Isovaleric acid *p*-tolylester, R^2^ = 0.99849, IC_50_ = 80.34 ± 1.12 μM. (**D**) Linolenic acid, R^2^ = 0.99997, IC_50_ = 54.44 ± 0.98 μM.

The standard compounds were purchased and screened for activity by using the erythrocyte lysis model; the results are shown in Figure 4. The compounds with better activity were isovaleric acid *p*-tolylester and linoleic acid. Subsequently, the erythrocyte lysis model was again utilized to test IC_50_ of isovaleric acid *p*-tolylester and linoleic acid, which were 80.34 ± 1.12 μM and 54.44 ± 0.98 μM, respectively (Figure 5). However, the dose dependence of linoleic acid was relatively weak, potentially serving as the underlying reason for the weaker dose dependence exhibited by fraction 2. This weak dose dependency may be due to its interaction with the erythrocyte membrane, leading to hemolysis and resulting in a false positive outcome. Meanwhile, our group has developed a novel screening model for UT-B inhibitors in vitro, which significantly reduces false positive results caused by hemolysis. In subsequent publications, we will report on this model in detail. Therefore, isovaleric acid *p*-tolylester was used in the subsequent study.

In prior research, Lu et al. [29] performed GC-MS analysis on celery seed essential oil and identified isovaleric acid *p*-tolylester at a concentration of 14.94%. Additionally, Senzosenkosi et al. [43] detected the same compound in the methanol extract of *Pleurotus ostreatus* mushrooms cultivated on sugarcane using GC-MS. Currently, it can be used both as a flavoring agent and a food preservative [44]. Regrettably, there are limited studies on the pharmacological activity of isovaleric acid *p*-tolylester. This study is the first to demonstrate that isovaleric acid *p*-tolylester exhibits UT-B inhibitory activity in vitro, providing a new perspective for research on its pharmacological effects.

### 2.4. Molecular Docking Analysis

**Figure 6:**
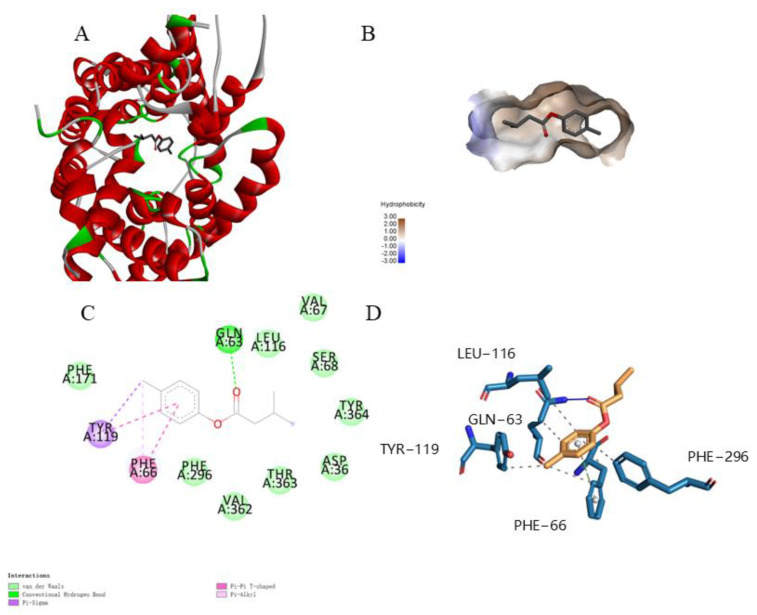
Docking model of isovaleric acid *p*-tolylester and UT-B. (**A**) The computational model of isovaleric acid *p*-tolylester bound to UT-B. (**B**) The binding pocket of isovaleric acid *p*-tolylester and UT-B. (**C**) The 2D plot of UT-B interaction with isovaleric acid *p*-tolylester. (**D**) The interaction between UT-B and isovaleric acid *p*-tolylester.

To study the binding interaction between the UT-B protein and Isovaleric acid *p*-tolylester, the molecular docking model was applied. Due to the presence of a known small molecule ligand, selenourea, in the crystal structure of the Bos Taurus UT-B urea transporter (PDB ID: 4EZD) that binds to selenourea, we performed molecular docking studies at the positional structure of selenourea, and the UT-B protein structure is a trimeric, so we chose one of the chains (A chain) for this study. The isovaleric acid *p*-tolylester can be bound to a hydrophobic pocket in the UT-B protein (Figure 6A,B). The isovaleric acid *p*-tolylester fitted well into this hydrophobic pocket, with its ester group forming a hydrophilic interaction with the side chain GLN: 63, its 4-methylphenyl forming Pi–Sigma and Pi–Alkyl interactions with the side chains TYR: 119 and PHE: 66, respectively, and its phenyl forming Pi–Pi T-shaped interactions with the side chains TYR: 119 and PHE: 66 (Figure 6C,D). Moreover, we compared the binding energies of isovaleric acid *p*-tolylester and selenourea to UT-B, with isovaleric acid *p*-tolylester (−6.8 kcal/mol) demonstrating superior binding compared to selenourea (−2.8 kcal/mol). This indicates that the binding of isovaleric acid *p*-tolylester to UT-B is more robust than that of selenourea to UT-B.

### 2.5. Establishment and Validation of the Common-Feature-Based Pharmacophore (HipHop)

Ligand-based pharmacophores allow for the discovery of lead compounds when the structure of the biological target is unknown by studying the structural information of the ligand that binds specifically to the target. By selecting five reported UT-B inhibitors with different scaffold structures, which are listed in Table 3, these five inhibitors were used as a training set for constructing the pharmacophore.

Table 4 lists the properties of the generated pharmacophore models and their scores, where all Direct Hits were 11111, all Partial Hits were 0000, and the Max Fit was four. Theoretically, pharmacophore models with higher scores have better properties; however, the best pharmacophore models still need to be validated. Therefore, additional UT-B inhibitors (Table 5) were used as a test set to validate the pharmacophore of 10 generated from the training set.

**Figure 7:**
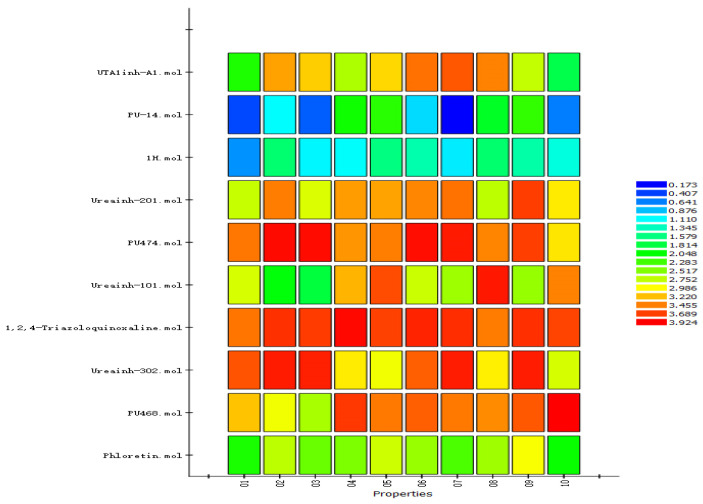
The test set and ligand-based pharmacophore mapping; all mappings are marked with different colors, and high scores refer to good fits.

**Figure 8:**
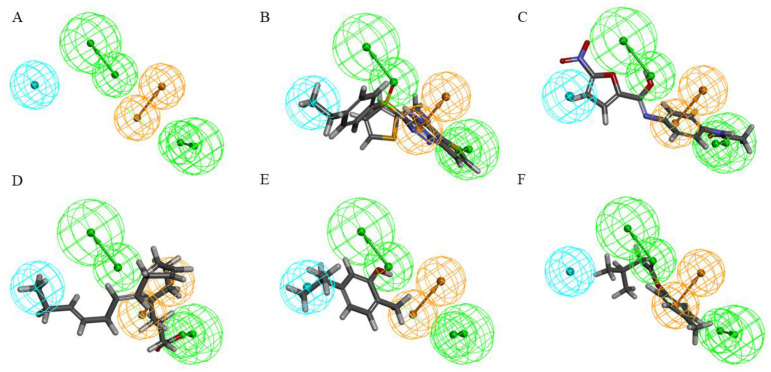
(**A**) Pharmacophore model (Hypo 8) of human UT-B inhibitors generated through H ipHop. (**B**) Hypo 8 mapping with compound UTBinh-14; (**C**) Hypo 8 mapping with compound **1H**; (**D**) Hypo 8 mapping with compound linolenic acid; (**E**) Hypo 8 mapping with compound carvacrol; (**F**) Hypo 8 mapping with compound isovaleric acid *p*-tolylester. The “Maximum Omitted Features” were set to −1.

Based on the matching of compounds to pharmacophores in the additional 10 test sets (Figure 7), Hypo 8 showed a better match. Using the UT-B selective inhibitor UTBinh-14 and the UT inhibitor 1H, we matched them with linolenic acid and carvacrol, which lack UT-B inhibitory activity, against the pharmacophore model. Both UTBinh-14 and 1H demonstrated good matching (Figure 8B,C), while linolenic acid and carvacrol showed poor matching (Figure 8D,E). This indicates that this model can also be used to evaluate the compounds in the present study. Hypo 8 consists of two hydrogen bond acceptors, a ring aromatic, and a hydrophobic center (Figure 8A). The compound has a good match with a hydrogen bonding receptor and a directional ring of the Hypo 8 model, and it has no match with the more distant hydrophobic and hydrogen bonding receptor, which suggests to us that this compound has room for subsequent structural modifications (Figure 8F).

### 2.6. Assessment of UT-B with Isovaleric Acid p-tolylester by Using MDs Simulation

**Figure 9:**
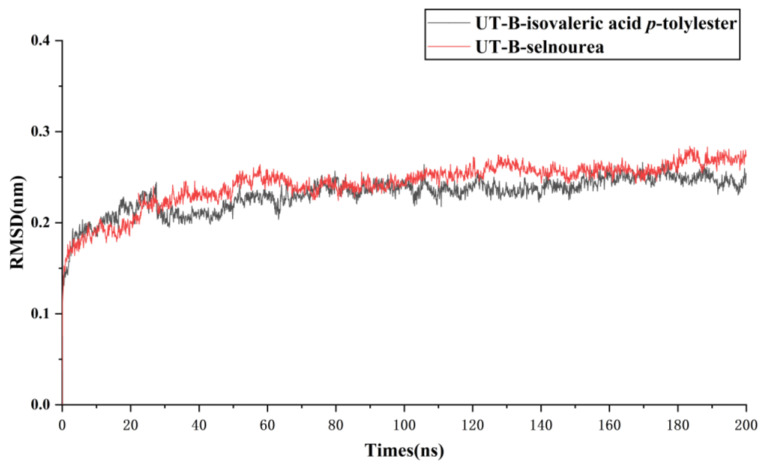
Time-dependent RMSD of modeled Bos Taurus bound to Selenourea UT-B with isovaleric acid *p*-tolylester in 200 ns of MDs simulations.

The stability of protein binding to small molecule ligands can be shown through the molecular dynamics simulation (MDs) process. Thus, 200 ns MDs simulation was used to assess the binding stability of UT-B protein to isovaleric acid *p*-tolylester. The fluctuations of RMSD values are shown in Figure 9. The whole system exhibited a mean RMSD of 0.23 ± 0.02 nm and became stable after 74.5 ns of simulation. After 100 ns, the isovaleric acid *p*-tolylester-UT-B co-crystal exhibited lower RMSD values than the selnourea-UT-B co-crystal, indicating that the ligand–protein binding ability of the former was stronger, and, consequently, the whole system remained stable.

**Figure 10:**
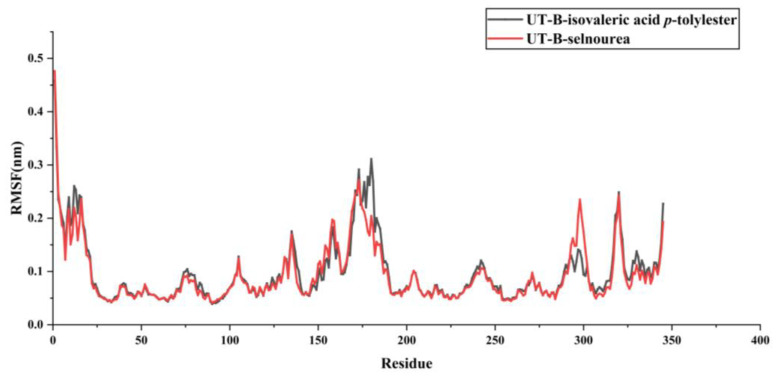
RMSF analysis of MD simulations for protein–ligand complexes.

In the UT-B complexes (Figure 10), the root mean square fluctuation (RMSF) curves for the UT-B-isovaleric acid *p*-tolylester and UT-B-selenourea complexes show considerable overlap, with both complexes exhibiting minimal fluctuations in amino acid residues between positions 80 and 90. The RMSF values for both complexes remain below 0.45 nm, indicating that the binding of the compounds does not induce significant fluctuations in the amino acid residues of UT-B. This suggests a high degree of stability for both complexes.

**Figure 11:**
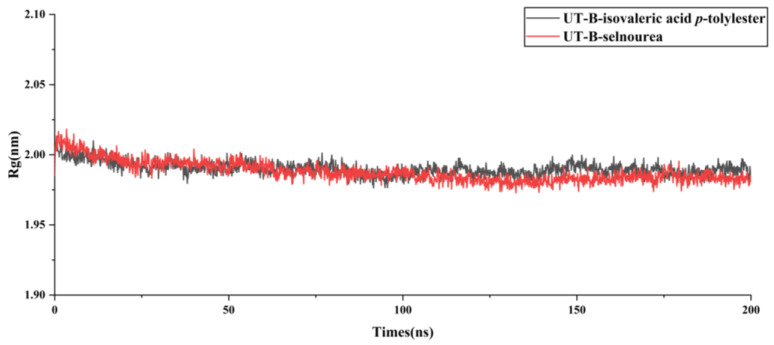
Rg analysis of MD simulations for protein–ligand complexes.

The radius of gyration (Rg) measures the compactness of a complex structure, with larger Rg values indicating expansion and smaller values suggesting a more compact system. In the UT-B–ligand complexes (Figure 11), the Rg curves for both compound complexes overlap closely with fluctuations maintained within the range of 1.95–2.05 nm. These findings indicate that the UT-B forms compact and stable complexes with both the co-crystallized ligand and isovaleric acid *p*-tolylester.

### 2.7. SwissADME Prediction of Isovaleric Acid p-tolylester

The data predicted for the physicochemical properties, lipophilicity, water solubility, pharmacokinetics, drug likeness, and medicinal chemistry of isovaleric acid *p*-tolylester evaluated through SwissADME are given in Table S2. According to Lipinski’s rule of five, the molecular weights of isovaleric acid *p*-tolylester were 192.25 g/mol within the limit of less than 500. The logP values were less than 5 and in the range of 2.70–3.54, which indicates that isovaleric acid *p*-tolylester possesses favorable ADME properties, which holds significant guiding value for subsequent research.

## 3. Materials and Methods

### 3.1. Chemicals, Drugs, and Reagents

The celery seeds were purchased from Xinjiang HEJI Traditional Chinese Medicine Decoction Pieces Co., Ltd. The plant material was identified by Professor Yun Zhu (School of Pharmaceutical Sciences, Shihezi University). Phloretin, acetamide glucose, NaCl, carvacrol, Cinene, Valeric acid, Allyl phenyl ether, *p*-Hydroxybenzaldehyde, linolenic acid, *β*-Myrcene, alcohol, dichloromethane, and methanol were all purchased from Energy^®^ (Shanghai, China). Isovaleric acid *p*-tolylester was purchased from TCI^®^ (Shanghai, China).

### 3.2. Preparation of Extracts

The seeds of *Apium graveolens* L. were finely ground and sieved through a 60-mesh screen. The resulting powder was then soaked in a 95% ethanol solution. The mixture was subjected to reflux heating for 3 h. After initial heating, the supernatant was carefully decanted. Additional 95% ethanol was added to the residue, and the reflux heating process was repeated. This extraction procedure was carried out a total of three times. The supernatants from each extraction were pooled together. The combined supernatant was then distilled and concentrated under reduced pressure to yield the alcohol extract of celery seed. Then, gradient elution using a dichloromethane and methanol system was employed to separate the alcohol extract of celery seeds through silica gel column chromatography. The gradient elution was performed with dichloromethane:methanol = 100:0 to dichloromethane:methanol = 0:100, resulting in 10 fractions of varying polarity. Finally, the extracts were stored in sealed vials at 4 °C for further use.

### 3.3. Establishment of a Compound Library for Celery Seeds

Using resources like Scifinder, Chemicalbook, and other relevant databases, the literature on the chemical composition of celery seeds was reviewed and summarized to create a celery seed compound library. The compound library of celery seeds contains a total of 119 compounds, which can be classified into flavonoids, volatile oils, fatty acids, and other compounds (S1).

### 3.4. Animals

Male Sprague-Dawley (SD) rats weighing 250~300 g were purchased from Xinjiang Medical University. The animals were housed in the animal laboratory of Shihezi University at a room temperature of 22 ± 2 °C with free access to food and water. The experiments were approved by the Ethics Committee of the First Affiliated Hospital, School of Medicine, Shihezi University (protocol code A2025-198).

### 3.5. Collection of Rat Blood and Erythrocyte Suspension Preparation

Rat whole blood was collected through orbital puncture and collected in tubes containing heparin sodium solution, which were then slightly shaken to mix the plasma with the anticoagulant uniformly. When fully mixed, erythrocyte suspensions were centrifuged at a speed of 1200 rpm. Erythrocyte were collected at the bottom of tubes and diluted to hematocrit of 1% in PBS containing acetamide (1.25 M) and glucose (5 μM). The tubes with erythrocyte were stored at 4 °C and used within 48 h.

### 3.6. UT-B Inhibitory Activity

The inhibitory activity of compounds was assayed by using erythrocyte lysis assays [48]. We configured 1% erythrocyte suspension, which contains 1.25 M acetamide and 5 mM glucose. The assayed compounds were prepared in two concentrations of 25 μM and 100 μM, and phloretin as a positive drug was used as a control. A vessel containing 99 μL of the erythrocyte suspension was added with 1 μL (10 μM final compound concentration, 1% final DMSO concentration) of the assayed compound and incubated for 20 min, and then 20 μL was added to the isotonic buffer. Measurements of erythrocyte lysis were carried out by using a microplate spectrophotometer (Cytation 3). The absorbance value was measured at 710 nm and finished within 5 min. The %lysis = 100% × (A_neg−_A_test_)/(A_neg−_A_pos_), where A_test_ is the absorbance value from a test well. Each group were measured three times repeatedly.

### 3.7. UPLC-TOF-MS Analysis

The chromatography analysis was performed on an ACQUITY UPLC^®^ BEH C18 (1.7 μm, 2.1 mm × 100 mm); the flow rate was set to 0.2 mL/min; the injection volume was 1.0 μL; the column temperature was set to 30 °C; and the detector wavelength was 254 nm. The mobile phase gradient elution conditions are shown in Table 6, which consisted of solvent A (methanol) and solvent B (0.1% formic acid). The mass spectrometric measurement was performed with electrospray ionization (ESI), and the ion modes were positive and negative interaction mode, with an ion source temperature of 100 °C. The desolvent gas was nitrogen at a flow rate of 450 L/h and a temperature of 200 °C in positive and negative ionization modes. In both positive and negative ionization modes, the capillary voltage was 2.1 kV, the cone pore voltage was 100 V, and the molecular weight scanning range was 100–1400 Da. The accurate mass number was determined using leucine enkephalin. The mass number was corrected using leucine enkephalin, and the ^13^C isotope peak (A+1)^+^ was chosen at a concentration of 200 pg/mL; the exact mass number was 557.2802, and the exact mass number was 0.599. The UPLC-TOF-MS system was operated with the software MassLynx 4.1.

### 3.8. Molecular Docking

Molecular docking was performed using Autodock 4.2 software [50]. Autodock 4.2 finds the global energy minima in the binding energy of a substrate and a target protein by combining a rapid grid-based energy evaluation with an efficient search of torsional degrees of freedom approach to find global energy minima in the binding energies of substrate and target proteins by searching the possible degrees of freedom of the whole system.

First, the three-dimensional structures of the compounds were established. The 3D structures of these compounds were subsequently energy minimized through Gaussian 09. The crystal structures of the UT-B used for docking were obtained from the PDB database (http://www.rcsb.org, accessed on 22 November 2022), PDB ID 4EZD. The UT-B structure is trimeric, so the A chain of the subsequent UT-B receptor is used for docking calculations. Before performing the docking, the target protein was pretreated by removing the proto-ligand and repairing some amino acid residues. The coordinates of the proto-ligand (selnourea, x = 44.219, y = −17.576, z = 27.599) in the target protein were used as the center of the docking box, with a box size of 40*40*40, while the compounds were then flexibly docked to the target protein, and the final output conformation was 10, with the conformation with the lowest global energy used for the subsequent study.

The chemical structures of the compounds used for the docking study were obtained from the PubChem database (https://pubchem.ncbi.nlm.nih.gov/, accessed on 22 November 2022).

### 3.9. Pharmacophore Modeling

The pharmacophores module in Discovery Studio (xDiscovery Studio 2019 Client) software was used to build the pharmacophore model [17]. The Common Feature Pharmacophore Generation protocol was used to generate pharmacophore models [29], and the characteristics of hydrogen bond donor (D), hydrogen bond acceptor (A), ring aromatic (R), and hydrophobic (H) of specific functional groups were chosen through DS 2019. All chemical structures of the compounds used were obtained from the PubChem database. Maximum pharmacophore hypotheses were set to 10, and the Minimum Interfeature Distance was set to 0.5, while all the other parameters were set to default values.

### 3.10. Molecular Dynamics Simulations

Small molecule–target protein complexes with the lowest absolute binding free energy in molecular docking were selected for molecular dynamics simulations using the Gromacs2023 [51] software package for the Frog Hopping Newton Integral Algorithm, which is used for equilibrium kinetic integration. During the simulation, Amber99SB force field parameters were used to generate the protein topology, and the small molecule ligands were first calculated using Multiwfn [52] for RESP2(0.5) (http://sobereva.com/476, accessed on 16 March 2025) charge, followed by Sobtop (Version: 1.0) (http://sobereva.com/soft/Sobtop, accessed on 16 March 2025) software to generate the GAFF force field topology. The TIP3P water model was used to add solvent to the protein–ligand system. Then, cubic boxes were built and supplemented with Na^+^/Cl^−^ to equilibrate the system. The energy of the complex system was optimized using the 50,000-step most rapid descent method. After system energy optimization was completed, the system temperature was steadily increased from 0 K to 300 K at a fixed volume and a constant heating rate. Then, 100 ps NVT (isothermal and isovolumetric) system simulations were performed to uniformly distribute the solvent molecules in the solvent at a system-sustaining temperature of 300 K. The system was then optimized through the most rapid descent method at 50,000 steps to equilibrate the system. Then, a 100 ps NPT (isothermal and isobaric) system simulation was performed for the composite system in one atmosphere. The simulation was performed with a time step of 2 fs, and the simulation trajectory was saved every 100 ps. Finally, a 200 ns molecular dynamics simulation was performed, and the trajectories were used to analyze the root mean square deviation (RMSD). Each group was measured three times repeatedly.

### 3.11. ADME Prediction

ADME prediction was performed to predict molecular properties using the SwissADME online server (http://www.swissadme.ch/, accessed on 10 March 2025). The molecular volume (Mv), molecular weight (Mw), logarithm of partition coefficient (milog P), number of hydrogen bond donors (HBDs), number of hydrogen bond acceptors (HBAs), topological polar surface area (TPSA), number of rotatable bonds (Nrotbs), and Lipinski’s rule of five of the synthesized compounds were determined.

### 3.12. Statistical Analysis

Statistical analysis was performed using Graphpad Prism 8.0.2 software. All of the data are expressed as means ± SEM. Statistical analysis was performed using Student’s *t*-test and one-way ANOVA followed by Fisher’s least significant difference analysis for multiple comparisons. A *p*-value less than 0.05 was considered statistically significant.

## 4. Conclusions

As a traditional Chinese medicine, celery seed (*Apium graveolens* L.) is widely used in the treatment of hypertension, ascites, and other diseases, However, current research on its mechanism of action has primarily focused on NBP. Nevertheless, the mechanism of the diuretic effect of celery seeds in Uyghur medicine and the identification of its active compounds remain unclear. Therefore, we are targeting urea transporter proteins to search for other substances in celery seed extract that can produce new pharmacological activities by inhibiting the activity of these urea transporter proteins. And, we provide a new research direction for studying the effects of celery seeds.

In this study, we screened the in vitro activities of different fractions of celery seed through erythrocyte lysis modeling and then utilized the UPLC-TOF-MS technique to determine their chemical compositions. We then screened these compounds for in vitro activities again. What is surprising is that the isovaleric acid *p*-tolylester was found to exhibit good inhibitory activity (IC_50_ = 80.34 μM) in vitro. Then, by combining computer techniques, such as molecular dynamics simulation and pharmacophore modeling, we investigated the mechanism of action of isovaleric acid *p*-tolylester, which has been found to have better in vitro inhibitory activity. Based on computer simulations, this experiment revealed that isovaleric acid *p*-tolylester could form a hydrophobic cavity with the UT-B protein and interact with multiple amino acid residues within this cavity, resulting in stable binding. Furthermore, according to the 200 ns molecular dynamics simulation, its binding mode was more stable than that of the UT-B–selnourea complex. In addition, when combined with pharmacophore modeling, isovaleric acid *p*-tolylester also exhibits good binding to the pharmacophore established in this experiment. And, through ADME predictions, isovaleric acid *p*-tolylester carbonate conforms with Lipinski’s rule of five, with its LogP value falling within the specified range, and it exhibits good water solubility. All of the above indicates that it has good drug-like properties. Based on our findings, we believe that isovaleric acid *p*-tolylester has great potential to inhibit UT-B, thereby exerting diuretic effects.

This study focused on celery seeds, a diuretic commonly used by Uyghur doctors, as the research subject and discovered a novel UT-B inhibitor through screening in vitro. Compared to previous research that screened UT-B inhibitors from small molecule libraries, natural products offer a broader range of scaffold structures, superior bioavailability, more abundant resources, and potentially fewer side effects. Currently, there is limited research on screening UT-B inhibitors from natural products. And, compared to the reported UT-B inhibitors, isovaleric acid *p*-tolylester exhibits more novel structural features, offering more possibilities for the subsequent development of new UT-B inhibitors. In the future, we will conduct structural modifications based on the molecular docking results of isovaleric acid *p*-tolylester to discover lead compounds with better activity. This will promote the development of UT-B inhibitors and contribute to the advancement of novel diuretic medications.

Screening for novel urea transporter inhibitors from traditional Chinese medicinal herbs can contribute to the development of these herbs and provide a new direction for the advancement of novel UT inhibitors.

## Figures and Tables

**Table 1:** Compounds identified from fFraction 1 throughby UPLC-TOF-MS.

Peak	Retention Time	Formula	[M+H]^+^	[M+H]^+^ Calculated	Error (ppm)	Compound
1	6.91	C_12_H_16_O_2_	193.1226	193.1229	−1.6	Isovaleric acid *p*-tolylester
2	7.03	C_10_H_14_O	173.0942	173.0942	0.0	Carvacrol
3	8.54	C_12_H_16_O_2_	385.2386 *	385.2379 *	1.8	L-3-n-butyl-4,5-dihydrophth-alide
4	8.56	C_15_H_24_	205.1957	205.1956	0.5	*β*-selinene

Note: * means [2M+H]^+^.

**Table 2:** Compounds identified from fraction 2 through UPLC-TOF-MS.

Peak	Retention Time	Formula	[M+H]^+^	[M+H]^+^ Calculated	Error (ppm)	Compound
1	3.64	C_10_H_16_	273.2582 *	273.2583 *	0.4	Cinene
2	3.90	C_5_H_10_O_2_	227.1260 *	227.1259 *	0.4	Valeric acid
3	3.89	C_9_H_10_O	291.1361 *	291.1361 *	0.0	Allyl phenyl ether
4	5.92	C_7_H_6_O_2_	267.0638 *	267.0633 *	1.9	*p*-Hydroxybenzaldehyde
5	6.98	C_10_H_14_O	173.0942	173.0942	0.0	Carvacrol
6	8.11	C_18_H_30_O_2_	279.2327	279.2324	1.1	Linolenic acid
7	8.50	C_10_H_16_	273.2586 *	273.2583 *	0.4	*β*-Myrcene

Note: * means [2M+H]^+.^

**Table 3:** Chemical structures of UT-B inhibitors collected as a training set.

NO.	Name	Structural	IC_50_/EC_50_
1	UTBinh-14 [45]	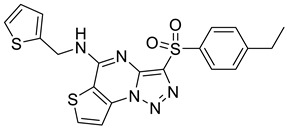	0.025 μM
2	PU168 [9]	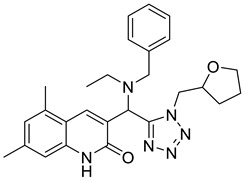	0.2 μM
3	PU-1424 [17]	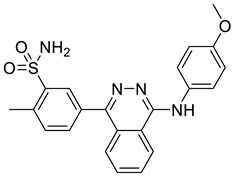	0.69 μM
4	25a [20]	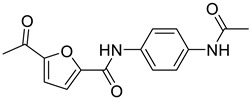	0.58 μM
5	CB-20 [46]	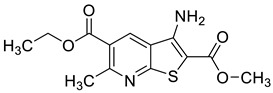	2.01 μM

**Table 4:** Common feature pharmacophore generation (HipHop).

No.	Features	Rank
1	RHAA	46.204
2	RHAA	46.204
3	RHAA	44.155
4	RHAA	44.155
5	RHAA	42.657
6	RHAA	42.090
7	RHAA	41.997
8	RHAA	41.102
9	RHAA	41.016
10	RHAA	40.861

**Table 5:** Chemical structures of UT-B inhibitors collected as a test set.

NO.	Name	Structural	IC_50_/EC_50_
1	Phloretin [47]	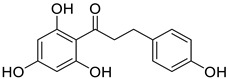	0.77 mM
2	PU468 [9]	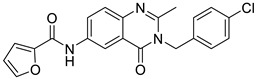	0.80 μM
3	1H [19]	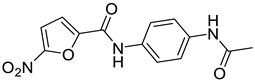	1.60 μM
4	PU-14 [15]	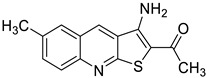	0.2 μM
5	PU-474 [9]	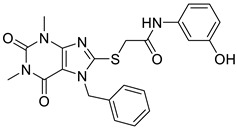	0.28 μM
6	Ureainh-101 [48]	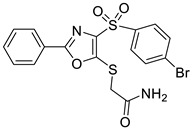	0.03 μM
7	Ureainh-201 [48]	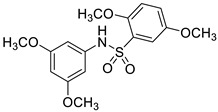	0.3 μM
8	Ureainh-302 [48]	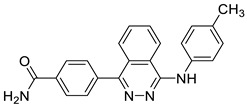	0.2 μM
9	1,2,4-Triazoloquinoxaline [18]	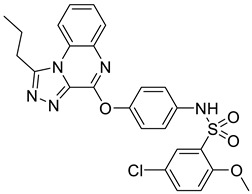	2 μM
10	UTA1inh-A1 [49]	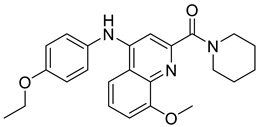	16 μM

**Table 6:** Mobile phase gradient elution conditions for samples.

Time	A (%)	B (%)	Curve
0	10	90	
1.50	35	65	6
5.50	50	50	6
8.00	75	25	6
12.50	97	3	6
14.50	97	3	6
15.00	10	90	6
17.00	10	90	6

## Data Availability

The data presented in this study are available in the article.

## Supplementary Materials

The following supporting information can be downloaded at https://www.mdpi.com/article/10.3390/molecules30071540/s1, Table S1. Compound Library for Celery Seeds; Table S2: physicochemical properties, lipophilicity, water solubility, pharmacokinetics, druglikeness, and medicinal chemistry of isovaleric acid *p*-tolylester predicted using SwissADME.
