# Supplementary material for: Identification and Validation of Urea Transporter B Inhibitor from Apium graveolens L. Seeds In Vitro and In Silico
Source: Molecules. 2025 Mar 30;30(7):1540. doi: 10.3390/molecules30071540 (PMC11990171; doi:10.3390/molecules30071540)
Supplement: Supplementary file 1 [file molecules-30-01540-s001.zip › molecules-3512194-supplementary.pdf]

Table S1. Compound Library for Celery Seeds.

No.	Name	Formula	Structure	Ref.
1	*p*-Hydroxybenzaldehyde	$C_7H_6O_2$	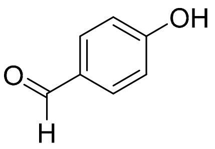	[1]
2	3-hydroxy-4-isopropyl-Benzoic acid	$C_{11}H_{14}O_3$	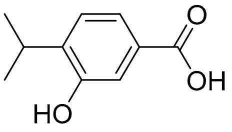	[1]
3	Vanillic acid	$C_9H_{11}O_4$	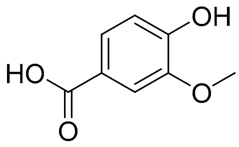	[1]
4	4-hydroxy-2-isopropyl-5-methyl-Phenyl-1-O-*β*-D-Glucopyranoside	$C_{16}H_{24}O_7$	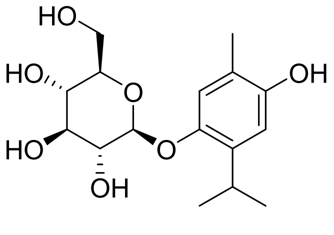	[1]
5	luteolin-7-O-*β*-D-glucopyranoside	$C_{21}H_{18}O_{12}$	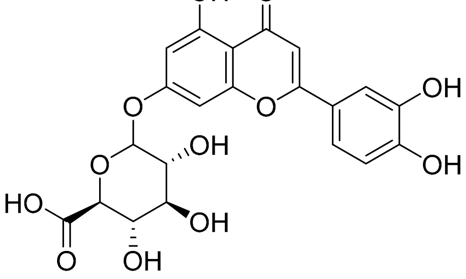	[2]

No.	Name	Formula	Structure	Ref.
6	luteolin-3'-O-*β*-D-glucopyranoside	$C_{21}H_{18}O_{12}$	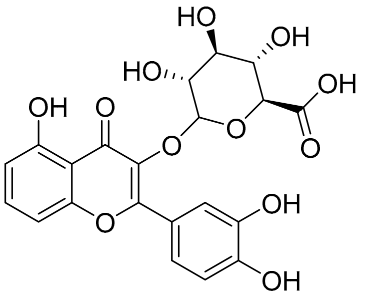	[2]
7	diosmetin-7-O-β-D-glucopyranoside	$C_{22}H_{22}O_{11}$	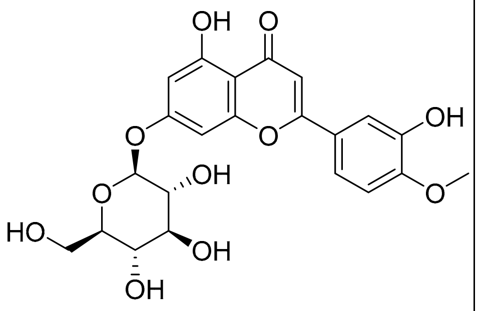	[2]
8	chrysoeriol	$C1_{6}H_{12}O_{6}$	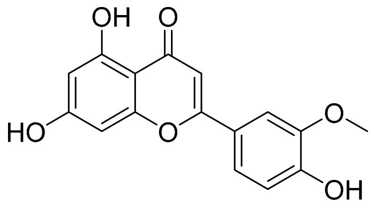	[2]
9	Cinene	$C_{10}H_{16}$	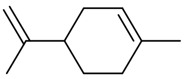	[3]
10	*β*-selinene	C15H24	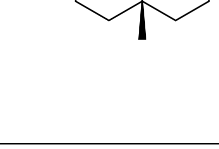	[3]

No.	Name	Formula	Structure	Ref.
11	Isovaleric acid *p*-tolylester	$C_{12}H_{16}O_2$	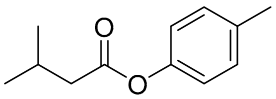	[3]
12	(+)-longifolene	$C_{15}H_{24}$	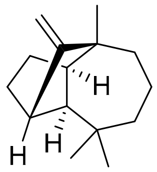	[3]
13	*β*-elemene	$C_{15}H_{24}$	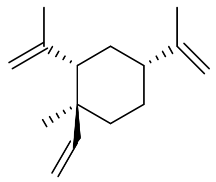	[3]
14	4-phenyl-1-buten-4-ol	$C_{10}H_{12}O$	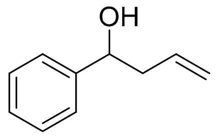	[3]
15	Pinocarvyl acetate	$C1_2H_{18}O_2$	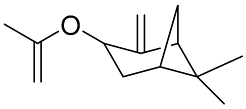	[3]

No.	Name	Formula	Structure	Ref.
16	Butylated hydroxytoluene	$C_{15}H_{24}O$	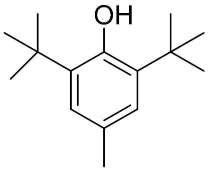	[3]
17	*β*-Myrcene	$C_{10}H_{16}$	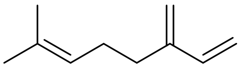	[3]
18	*γ*-Terpinene	$C_{10}H_{16}$	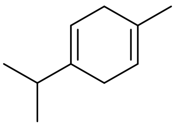	[4]
19	*O*-cymene	$C_{10}H_{14}$	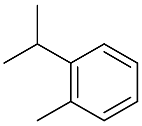	[4]
20	Carvacrol	$C_{10}H_{14}O$	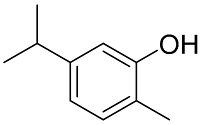	[4]

No.	Name	Formula	Structure	Ref.
21	Dibutyl phthalate	$C_{16}H_{22}O_4$	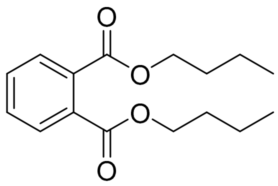	[4]
22	Butyl phthalyl butyl glycolate	$C_{18}H_{24}O_6$	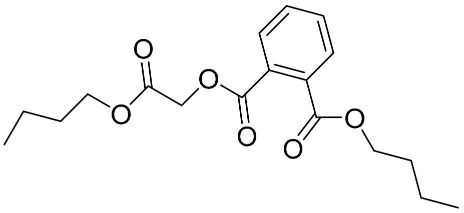	[4]
23	Butyl isobutyl phthalate	$C_{16}H_{22}O_4$	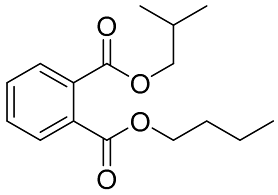	[4]
24	*α*-Thujenc	$C_{10}H_{16}$	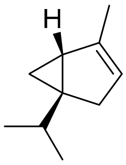	[4]
25	*β*-phellandrene	$C_{10}H_{16}$	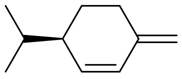	[4]

No.	Name	Formula	Structure	Ref.
26	*α*-terpinene	$C_{10}H_{16}$	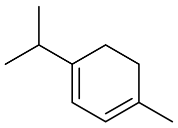	[4]
27	Sabinene	$C_{10}H_{16}$	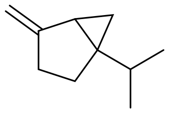	[4]
28	*β*-Eudesmol	$C_{15}H_{26}O$	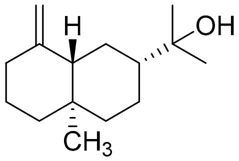	[5]
29	*Trans*-(-)-carveol	$C_{10}H_{16}O$	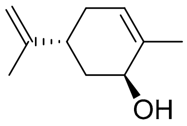	[5]
30	Linalool	$C_{10}H_{18}O$	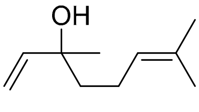	[5]

No.	Name	Formula	Structure	Ref.
31	Dihydro cuminyl alcohol	$C_{10}H_{16}O$	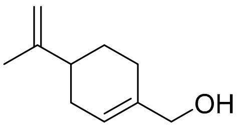	[5]
32	Lavandulol	$C_{10}H_{18}O$	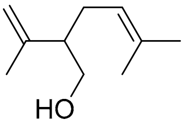	[5]
33	(-)-*Trans*-pinocarveol	$C_{10}H_{16}O$	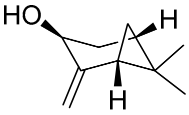	[5]
34	1-Octanol	C8H18O	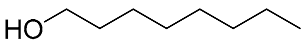	[5]
35	(-)-Perillaldehyde	$C_{10}H_{14}O$	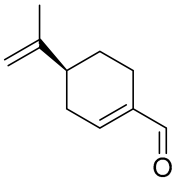	[5]

No.	Name	Formula	Structure	Ref.
36	Valerophenone	$C_{11}H_{14}O$	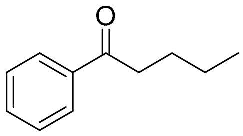	[5]
37	Carvone	$C_{10}H_{14}O$	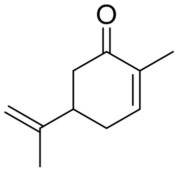	[5]
38	(+)-dihydrocarvone	$C_{10}H_{16}O$	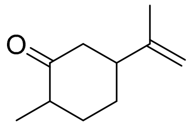	[5]
39	methyl *(1S,3S,5S)*-3-hydroxy-6,6-dimethyl-2-methylenebicyclo[3.1.1]heptane-1-carboxylate	$C_{12}H_{18}O_3$	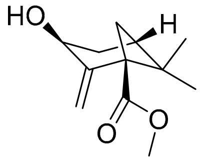	[5]
40	1-Octen-3-yl acetate	$C_{10}H_{18}O_2$	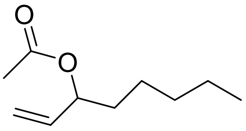	[5]

No.	Name	Formula	Structure	Ref.
41	(-)-Dihydrcocarvyl acetate	$C_{12}H_{20}O_2$	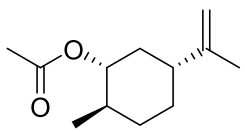	[5]
42	3-Octyk aceate	$C_{10}H_{20}O_2$	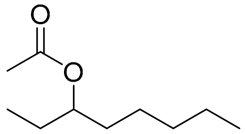	[5]
43	*α*-Selinene	$C_{15}H_{24}$	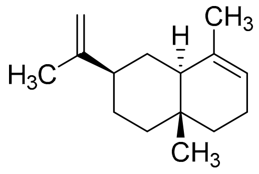	[5]
44	ALPHA-PINENE	$C_{10}H_{16}$	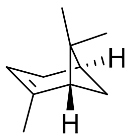	[5]
45	*γ*-Selinene	$C_{15}H_{24}$	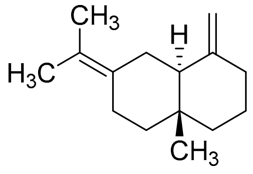	[5]

No.	Name	Formula	Structure	Ref.
46	*α*-pinene	$C_{10}H_{16}$	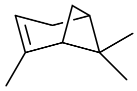	[5]
47	(-)-*β*-caryophyllene	$C_{15}H_{24}$	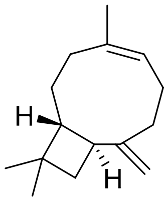	[5]
48	β-Elemene	$C_{15}H_{24}$	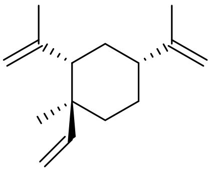	[5]
49	Terpinolene	$C_{10}H_{16}$	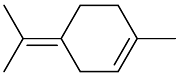	[5]
50	Valenciene	$C_{15}H_{24}$	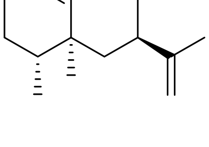	[5]

No.	Name	Formula	Structure	Ref.
51	3-Carene	$C_{10}H_{16}$	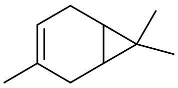	[5]
52	*α*-bulnesene	$C_{15}H_{24}$	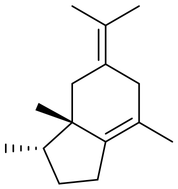	[5]
53	1-Methyl-4-(1-methylethyl)-1,3-cyclohexadiene	$C_{10}H_{16}$	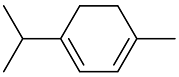	[5]
54	*γ*-Elemene	$C_{15}H_{24}$	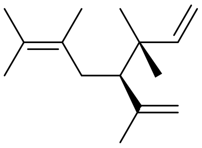	[5]
55	Comphene	$C_{10}H_{16}$	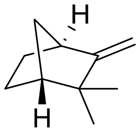	[5]

No.	Name	Formula	Structure	Ref.
56	(+)-Limonene oxide	$C_{10}H_{16}O$	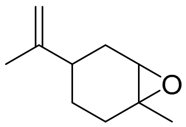	[5]
57	*(E)*-3,7-dimethylocta-1,3,6-triene	$C_{10}H_{16}$	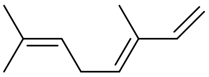	[5]
58	Valeric acid	$C_5H_{10}O_2$	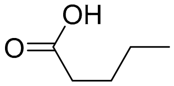	[5]
59	Octanoic acid	$C_8H_{16}O_2$	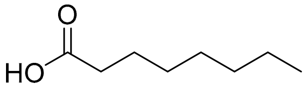	[5]
60	3-*n*-butylphenylhydrazine	$C_{10}H_{16}N_2$	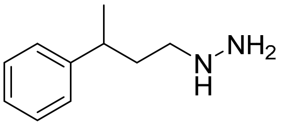	[5]

No.	Name	Formula	Structure	Ref.
61	Caryophyllene oxide	$C_{15}H_{24}O$	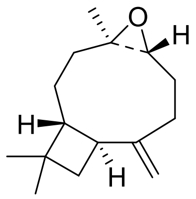	[5]
62	*p*-Dimethylstyrene	$C_{10}H_{12}$	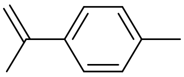	[5]
63	*cis*-Anethol	$C_{10}H_{12}O$	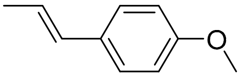	[5]
64	(+)-Dipentene	$C_{10}H_{16}$	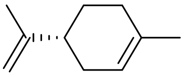	[6]
65	5-isopropyl-2-methylenecyclohexan-1-ol	$C_{10}H_{16}O$	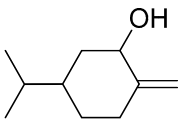	[6]

No.	Name	Formula	Structure	Ref.
66	*(1R,5R)*-5-isopropyl-2-methylcyclohex-2-en-1-ol	$C_{10}H_{18}O$	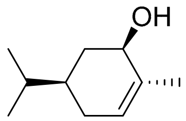	[6]
67	*β*-Caryophyllene	$C_{15}H_{24}$	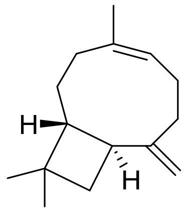	[6]
68	*α*-Caryophyllene	$C_{15}H_{24}$	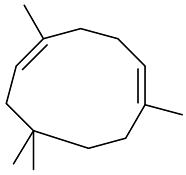	[6]
69	*α*-curcumene	$C_{15}H_{22}$	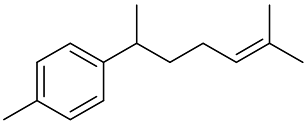	[6]
70	eudesma-4(14),11-diene	$C_{15}H_{24}$	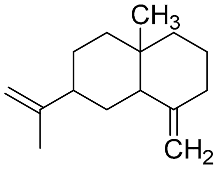	[6]

No.	Name	Formula	Structure	Ref.
71	3-*n*-Butyphthalide	$C_{12}H_{14}O_2$	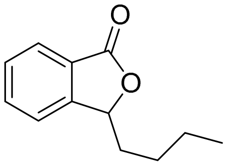	[7]
72	*L*-3-*n*-butyl-4,5-dihydrophthalide	$C_{12}H_{16}O_2$	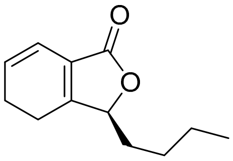	[7]
73	2-Methyl-1,4-butanediol	$C_5H_{12}O_2$	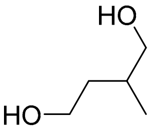	[8]
74	2-Methyl butyric acid	$C_5H_{10}O_2$	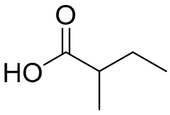	[8]
75	*o*-Xylene	C_8H_{10}	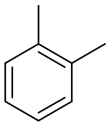	[8]

No.	Name	Formula	Structure	Ref.
76	(3*E)*-3-Undecen-5-yne	$C_{11}H_{18}$	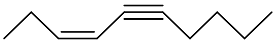	[8]
77	Phenylpentane	$C_{11}H_{16}$	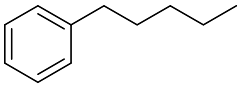	[8]
78	(+)-*trans*-*p*-Mentha-2,8-dien-1-ol	$C_{11}H_{16}O$	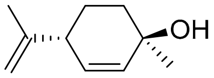	[8]
79	Allyl phenyl ether	$C_9H_{10}O$	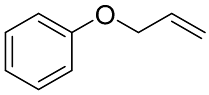	[8]
80	5-Methylindane	$C_{10}H_{12}$	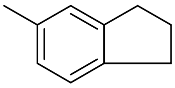	[8]

No.	Name	Formula	Structure	Ref.
81	Methylnaphthalene	$C_{11}H_{10}$	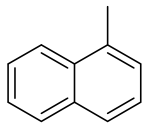	[8]
82	Farnesene	$C_{15}H_{24}$	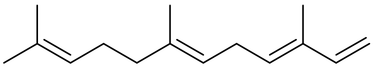	[8]
83	Octanophenone	$C_{14}H_{20}O$	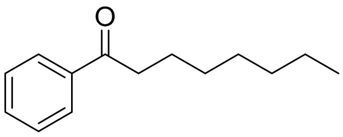	[8]
84	Biphenyl	$C_{12}H_{10}$	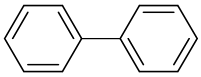	[8]
85	Tributyl phosphate	$C_{12}H_{27}O_4P$	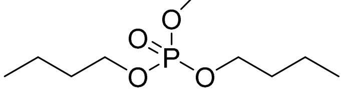	[8]

No.	Name	Formula	Structure	Ref.
86	5-Indanol	$C_9H_{10}O$	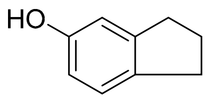	[8]
87	Allyl phenoxyacetate	$C_{11}H_{12}O_3$	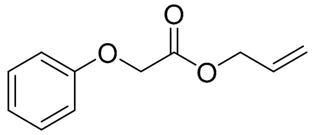	[8]
88	*n*-Hendecane	$C_{11}H_{24}$	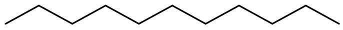	[8]
89	Bornyl acetate	$C_{12}H_{20}O_2$	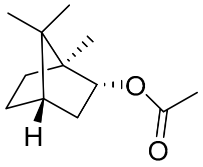	[8]
90	*β*-sesquiphellandrene	$C_{15}H_{24}$	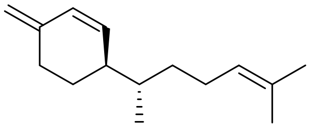	[8]

No.	Name	Formula	Structure	Ref.
91	Apiole	$C_{12}H_{14}O_4$	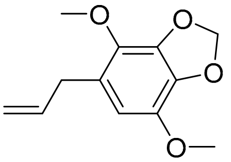	[8]
92	(+)-Lenene	$C_{15}H_{24}$	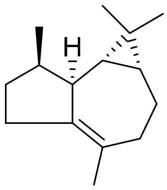	[8]
93	(*E*, *Z*)-Farnesol	$C_{15}H_{26}O$	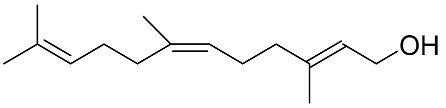	[8]
94	Palmitic acid ethyl ester	$C_{18}H_{36}O_2$	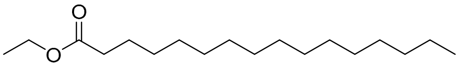	[8]
95	(*E*)-3-Eicosene	$C_{20}H_{40}$	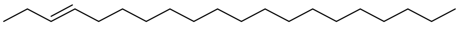	[8]

No.	Name	Formula	Structure	Ref.
96	Phenylpentane	$C_{11}H_{16}$	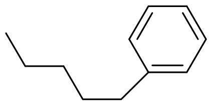	[8]
97	2-methyl-5-(1-methylethylidene)-Cyclohexanol	$C_{10}H_{18}O$	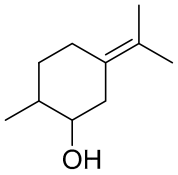	[8]
98	1-methyl-4-(propan-2-ylidene)-cyclohex-1-ene	$C_{10}H_{16}$	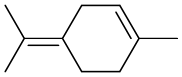	[8]
99	1-ethyl-2,4-diisopropyl-1-methylcyclohexane	$C_{15}H_{30}$	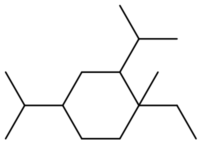	[8]
100	*(2R,3S)*-1,1-dimethyl-3-(pent-1-en-2-yl)-2-propylcyclobutane	$C_{15}H_{24}O$	isobutane 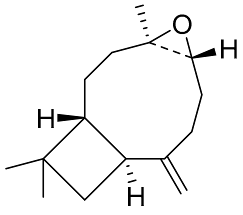	[8]

No.	Name	Formula	Structure	Ref.
101	Visnagin	$C_{13}H_{10}O_4$	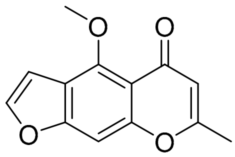	[8]
102	9*(E)*,12*(Z)*-Octadecadienoic acid methyl ester	$C_{19}H_{34}O_2$	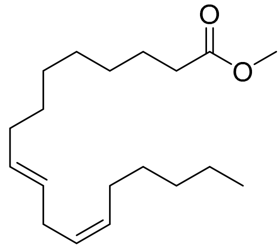	[8]
103	Ethyl oleate	$C_{20}H_{38}O_2$	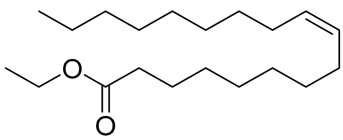	[8]
104	Bis(2-ethylhexyl) phthalate	$C_{24}H_{38}O_4$	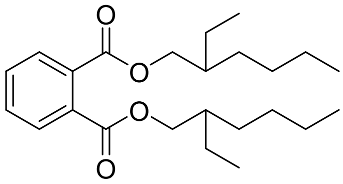	[8]
105	Sedanolide	$C_{12}H_{18}O_2$	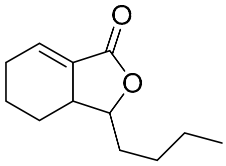	[7]

No.	Name	Formula	Structure	Ref.
106	Stearic acid	$C_{18}H_{36}O_2$	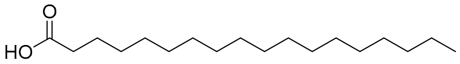	[9]
107	*Trans*-6-octadecenoate	$C_{19}H_{36}O_2$	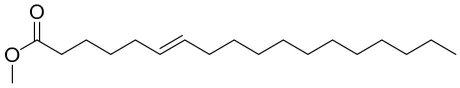	[6]
108	Methyl pentadecanoate	$C_{16}H_{32}O_2$	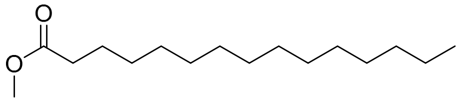	[6]
109	Methyl palmitate	$C_{17}H_{34}O_2$	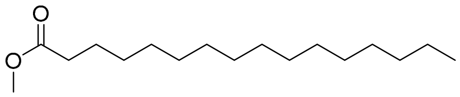	[6]
110	Palmitic acid	$C_{16}H_{32}O_2$	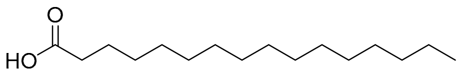	[6]

No.	Name	Formula	Structure	Ref.
111	Methyl octadecanoate	$C_{19}H_{38}O_2$	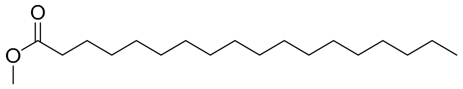	[6]
112	Methyl eicosanoate	$C_{21}H_{42}O_2$	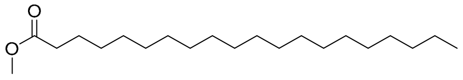	[6]
113	Linoleic acid	$C_{18}H_{32}O_2$	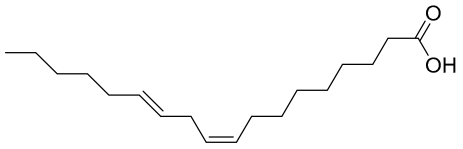	[6]
114	Linolenic acid	$C_{18}H_{30}O_2$	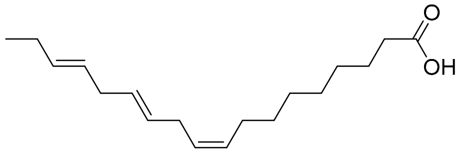	[6]
115	Oleic acid	$C_{18}H_{34}O_2$	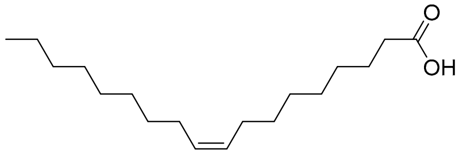	[6]

No.	Name	Formula	Structure	Ref.
116	Arachidic Acid	$C_{20}H_{40}O_2$	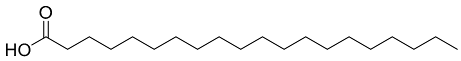	[6]
117	Palmitel acid methyl ester	$C_{19}H_{36}O_2$	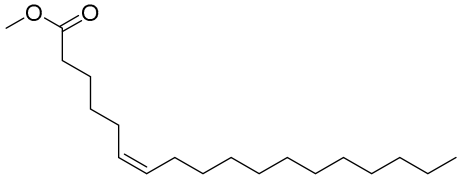	[6]
118	Plmitelaidic acid methyl ester	$C_{17}H_{32}O_2$	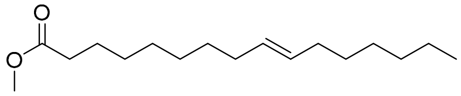	[6]
119	*L*-Glutamic acid	$C_5H_9NO_4$	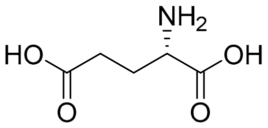	[10]

Table S2: physicochemical properties, lipophilicity, water solubility, pharmacokinetics, druglikeness, and medicinal chemistry of isovaleric acid *p*-tolylester predicted using SwissADME.

**Physicochemical Properties**	**Lipophilicity**	**Water Solubility**
Formula: $C_{12}H_{16}O_2$<br>Molecular weight: 192.25 g/mol<br>Num. heavy atoms: 14<br>Num. arom. heavy atoms: 6<br>Fraction Csp3: 0.42<br>Num. rotatable bonds: 4<br>Num. H-bond acceptors: 2<br>Num. H-bond donors: 0<br>Molar Refractivity: 57.33<br>TPSA: 26.30 Å^2	Log $P_{o/w}$ (iLOGP): 2.70<br>Log $P_{o/w}$ (XLOGP3): 3.54<br>Log $P_{o/w}$ (WLOGP: 2.95<br>Log $P_{o/w}$ (MLOGP): 3.13<br>Log $P_{o/w}$ (SILICOS-IT) : 3.08<br>Consensus Log $P_{o/w}$: 3.08	Log *S* (ESOL): -3.32<br>Solubility: 9.30e-02 mg/ml; 4.84e-04 mol/l<br>Class: Soluble<br>Log *S* (Ali): -3.78<br>Solubility: 3.21e-02 mg/ml; 1.67e-04 mol/l<br>Class: Soluble<br>Log *S* (SILICOS-IT): -3.70<br>Solubility: 3.80e-02 mg/ml; 1.98e-04 mol/l<br>Class: Soluble
**Pharmacokinetics**	**Druglikeness**	**Medicinal Chemistry**
GI absorption: High<br>BBB permeant: Yes<br>P-gp substrate: No<br>CYP1A2 inhibitor: No<br>CYP2C19 inhibitor: No<br>CYP2C9 inhibitor: No<br>CYP2D6 inhibitor: No<br>CYP3A4 inhibitor: No<br>Log K_p (skin permeation): -4.96 cm/s	Lipinski: Yes; 0 violation<br>Ghose: Yes<br>Veber: Yes<br>Egan: Yes<br>Muegge: No; 1 violation: MW<200<br>Bioavailability Score: 0.55	PAINS: 0 alert<br>Brenk: 1 alert: phenol_ester<br>Leadlikeness: No; 2 violations: MW<250, XLOGP3>3.5<br>Synthetic accessibility: 1.57

Reference

1. Lv, J.; Mou, x.; Wang, W.; Liao, L.; HajiAkber, A. Studies on chemical constituents of seeds of Apium graveolens L. *Lishizhen Medicine and Materia Medica Research* **2006**, *17*, 6-7.
2. Qu, C.; Wang, Y.; Zhang, B.; Lin, Z. Advances in pharmacology research of celery seed *China Journal of Traditional Chinese Medicine and Pharmacy* **2019**, *34*, 5295-5299.
3. Lu, Z.G.; Li, W.; Wang, P.J. Chemical Composition and Ability of Scavenging DPPH Radical of Essential Oil and Residue from the Celery Seed. *Advanced Materials Research* **2011**, *183-185*, 18-21.
4. Abulimiti, L.; Liu, L.; HajiA, A.; Liao, L. Study of the chemical constituents in the essential oil from celery seed. *Natural product research and development* **2004**, *16*, 36-37.
5. Zeng, Q.; Gong, R.; Yang, H.; Pang, Y. Analysis of Essential Oil from Hunan Celery Seeds by GC/MS Combined with Retention Index. *Traditional Chinese Drug Research & Clinical Pharmacology* **2016**, *27*, 677-680.
6. Zhang, J.; Wang, J.; Li, T.; Hui, R.; Hou, D. GC/MS Analysis of Volatile Components in Western Celery Seeds from the United States. *Food Science* **2002**, *23*, 225-227.
7. Chen, W.; Shen, G.; Chen, H. Higt purity preparation and inentification of three phthalide compounds from Apium gravolens. *Journal of Pharmaceutical Practice* **2017**, *35*, 138-140.
8. Guo, L.; Chen, T.; Chen, X.; Qu, L.; Lu, J.; Zhao, Y. Study on the chemical composition of volatile oil in the celery seed. *Henan science* **2003**, *21*, 728-729.
9. Destaillats, F.; Lipids, P.A.J. Base-catalyzed derivatization methodology for FA analysis. application to milk fat and celery seed lipid TAG. *Lipids* **2002**, *37*, 527-532.
10. Liu, H. Study on Extraction, Purification and Hypolipidemic Biological Function of Celery Seeds Oil. Doctor, Shenyang Agriculural University, 2009.
